# A Context-Aware Model to Provide Positioning in Disaster Relief Scenarios

**DOI:** 10.3390/s151025176

**Published:** 2015-09-30

**Authors:** Daniel Moreno, Sergio F. Ochoa, Roc Meseguer

**Affiliations:** 1 Department of Computer Science, Universidad de Chile, Beauchef 851, Santiago 8370459, Chile; E-Mail: dmoreno@dcc.uchile.cl; 2 Department of Computer Architecture, Universitat Politècnica de Catalunya, Jordi Girona Salgado 1, Barcelona 08034, Spain; E-Mail: meseguer@ac.upc.edu

**Keywords:** outdoor positioning, context-aware model, disaster relief efforts

## Abstract

The effectiveness of the work performed during disaster relief efforts is highly dependent on the coordination of activities conducted by the first responders deployed in the affected area. Such coordination, in turn, depends on an appropriate management of geo-referenced information. Therefore, enabling first responders to count on positioning capabilities during these activities is vital to increase the effectiveness of the response process. The positioning methods used in this scenario must assume a lack of infrastructure-based communication and electrical energy, which usually characterizes affected areas. Although positioning systems such as the Global Positioning System (GPS) have been shown to be useful, we cannot assume that all devices deployed in the area (or most of them) will have positioning capabilities by themselves. Typically, many first responders carry devices that are not capable of performing positioning on their own, but that require such a service. In order to help increase the positioning capability of first responders in disaster-affected areas, this paper presents a context-aware positioning model that allows mobile devices to estimate their position based on information gathered from their surroundings. The performance of the proposed model was evaluated using simulations, and the obtained results show that mobile devices without positioning capabilities were able to use the model to estimate their position. Moreover, the accuracy of the positioning model has been shown to be suitable for conducting most first response activities.

## 1. Introduction

Several countries in South America and the Asia-Pacific region are frequently affected by natural disasters, such as earthquakes, tsunamis, landslides, and volcano eruptions. When these extreme events put urban areas at risk, a first response process is usually triggered by firefighting companies deployed in the affected area. In disaster relief efforts, typical participants include firemen, police officers, medical personnel and government agencies, and sometimes military units [1]. Every organization is in charge of specific activities that range from search and rescue to securing the affected area. In particular, firefighters and military units play a crucial role, since they are usually trained to address these challenges.

Typically, electric energy is not available in the affected area, because the power distribution network is usually damaged or turned off for security reasons. Similarly, the infrastructure-based communication network is frequently damaged or collapsed; therefore the communication is mediated mainly by very high/ultra high frequency (VHF/UHF) radio systems [2]. In such a scenario, the most critical response activities should be performed during the first 72 h after the event (also known as the golden relief time), since after such a period the probability of rescuing people alive is very low [3,4]. These activities include search and rescue of victims, providing first aid to injured people, diagnosing the stability of physical infrastructure, establishing evacuation and re-entry routes, and securing the working area. These activities are interconnected and they are conducted by first responders belonging to several relief organizations. Coordination among these parties has a direct impact on the effectiveness of the initial response to a disaster.

Typically, command posts deployed in the field try to coordinate the local activities, but they usually fail due to the unavailability of digital communication support and geo-referenced information that would allow them to know the status of the first response process [5,6]. Therefore, improvisation becomes the common denominator in those scenarios [7,8], which reduces the effectiveness of this process.

Most research work done to address this coordination problem is focused on providing digital communication in the field in order to support interactions with first responders [5,9,10,11,12]. However, few studies have been done on increasing their positioning capability. Although most of the information used and updated as part of their activities is geo-referenced, only a portion of first responders deployed in the affected area have positioning capability.

Considering the reality of many developing countries (e.g., all of Latin America), where the most important first response organization (*i.e.*, the firefighters) is composed almost exclusively of volunteers, financed mainly by the community and typically with few resources to address these tasks, the proportion of people who could have positioning capability during a first response process is low. However, they usually have several outdated mobile computing devices given to them by the community, e.g., cellular phones and laptops, which opens up the possibility of increasing the positioning capability in this scenario.

In order to help address this challenge, we propose a context-aware positioning model that takes advantage of computing devices with positioning capability (e.g., those embedding GPS), in order to provide positioning to those who are not able to do it by themselves. The proposed model enables devices in outdoor scenarios to estimate their position based on information gathered from their direct context (*i.e.*, their environment), regardless of their positioning capabilities. Devices using this model could estimate their position by sharing context and positioning information with their neighbors. Thus, devices with positioning capabilities could choose the better suited positioning strategy (from a pool) considering the user’s current context; for example, choosing a positioning strategy that is less energy consuming than using the de facto strategy. The positioning information of these nodes could then be shared with neighboring devices with limited or no positioning capabilities, allowing them to estimate their position collaboratively.

The positioning model was evaluated using simulated outdoor environments, which were implemented over well-known communication and mobility models. The obtained results show that devices without positioning capabilities are able to gather positioning information from access points (e.g., command posts, communication antennas, and emergency vehicles parked in the field) and other devices in their vicinity, and then use this information to estimate their own positions, although not as accurately as with a direct positioning strategy like GPS. Moreover, the devices are able to determine which positioning strategies can be used in a certain environment, and choose the one that involves the least energy consumption.

The next section shows the related work. The proposed context-aware positioning model is detailed in Section 3. The simulation scenario and the experiments are specified in Section 4, and the results are shown and discussed in Section 5. Section 6 establishes the limitations of this proposal. Section 7 presents the conclusions and future work.

## 2. Related Work

Most positioning approaches commonly rely on communication between a target resource (*i.e.*, a mobile device) and a series of reference points with known positions. There are several proposals to perform positioning on mobile, heterogeneous outdoor environments. These works aim to estimate the position of the targets using individual or collaborative approaches, and they are usually focused on finding novel alternatives to GPS and improving the accuracy of the measurements. Although some of these works are able to reduce the error of a positioning estimation to the range of centimeters, they typically require an existing communication infrastructure and/or a source of energy to function properly, both of which are usually not available in disaster areas.

Shin *et al*. [13] propose a personal indoor/outdoor Wi-Fi Positioning System using received signal strength (RSS) from dense Wi-Fi access points (AP) dedicated to localization, oriented towards Android-based smartphones. Their method measures the RSS from each AP three times, calculating the mean value and comparing it to each training value. If the difference between the mean value and the training values is below a given threshold, the training value is withdrawn and the mean of filtered training values is calculated again. Finally, the mean value is compared with the database value and a proper location on the map is found with a proper scan time and threshold, thereby yielding a low error rate. Although this proposal is interesting and widely used, it requires a mix of both *ad hoc* elements and a pre-existent infrastructure that is not necessarily available in a disaster scenario.

Global Navigation Satellite Systems (GNNS) incorporate several satellite-based positioning systems, including GPS, GLONASS (GLObal NAvigation Satellite System), Galileo, and Compass [14]. In GNSS, the measurements obtained from a group include extra measurements, specifically the relative distances between the neighboring users. As a result, there are several factors that impact on the positioning accuracy, and cause problems for the performance analysis. Huang *et al*. [15] studied the multiplicative effect of various factors on the accuracy of GNSS collaborative positioning algorithms, using their proposed Collaborative Dilution of Precision model. Using such a model, they also analyze the performance of GNSS standalone positioning. Their results show that GNSS collaborative positioning accuracy is always higher than or equal to that of GNSS standalone positioning.

Zhang *et al*. [16] present an approach in which a database (DB) is trained using navigational data from users, utilizing different strategies to survey sensor-based navigation errors in indoor positioning. They combine different navigation trajectories moving in and out of a target building to create the DB, restricting the time length of available indoor navigation trajectories, *i.e.*, the trajectories from the last received GNSS signal before entering an indoor area, until the next GNSS signal is received after walking out of the target building. Their results showed that the errors in pedestrian navigation were reduced from 6.0 to 5.7 m during 5–10 min of indoor walking. This is an interesting study that combines outdoor and indoor positioning, providing a smooth transition between these two types of scenarios; however, it is focused on providing indoor positioning, and outdoor signals are used only to establish entry and exit points throughout the user’s indoor route.

Sahoo *et al*. [17] state that collaboration is highly essential for nodes to perform positioning efficiently in outdoor and indoor environments. They use beacon and anchor nodes with known positions to estimate the position of a target node in a distributive manner, employing localization algorithms that consider fading and shadowing effects. They show analytical methods that use probability distribution functions to find the probability of wrongly identifying a transmission arriving from a node with location information, reducing the localization error and thus providing more accurate positioning information to the target node. These researchers state that their algorithm works well even when only one beacon node is present. The algorithm performs calculations with low time complexity, which is suitable when memory and energy constraints are present. This work is very similar to our own, with the difference that the authors use probability distribution functions to determine viable neighbor signals, and their experiments utilize smaller scenarios (200 × 300 m^2^) and a greater numbers of nodes (250–500) in order to achieve acceptable accuracy levels.

Savarese *et al*. [18] developed a two-phase distributed algorithm for determining the positions of nodes in an *ad hoc* wireless sensor network. The algorithm assumes that some of the users know their positions (*i.e.*, they act as anchors). The anchors broadcast their positions to their neighbors, allowing the latter to estimate their positions with a certain degree of confidence, which improves iteratively with each new transmission from the anchors. This work provides collaborative positioning in outdoor environments to all participants of an *ad hoc* network, but, unlike our model, the beacon nodes’ position is transmitted to all nodes in the network and then improved iteratively, which could significantly increase the energy consumption of the process and overload the communication link.

Capkun *et al*. [19] propose a distributed, infrastructure-free positioning algorithm that uses distance between mobile nodes to build a relative coordinate system. In this algorithm the nodes’ positions are computed in two dimensions, thereby providing location information accurate enough to support basic positioning capabilities within the environment. The nodes know the position of their neighbors using the relative coordinate system, but they have no way to translate such a position to a geographic coordinate system, unless nodes with GPS capabilities are included in the network. This is an interesting proposal that enables nodes in an *ad hoc* network to collaboratively determine the positions of each neighbor in relation to their own; however, it does not take into account the physical scenario of the devices, and thus is unable to provide the actual geographical position of the nodes.

Shaw *et al*. [20] apply the problem of mapping an estimate of a user’s current location to a semantically meaningful point of interest, such as a home, restaurant, or store. To this end, they propose a spatial search algorithm that infers a user’s location by combining aggregate signals mined from billions of foursquare check-ins with real-time contextual information. This work uses machine learning techniques to create an optimal ranking function, which learns from large amounts of implicit feedback, evaluating the performance of their system in a variety of real-world situations. This novel work takes advantage of social networks’ positioning information to locate target devices; however, given the nature of the algorithms used, it requires an extensive location database that could be unavailable in disaster relief scenarios.

Eltahir [21] presents a study of the effect of some radio propagation models, such as free space and two-ray ground, on the communication capability of devices in simulated urban mobile *ad hoc* networks. The study shows that commonly used propagation models do not necessarily provide results similar to real-world conditions. Thus, choosing an incorrect propagation model could translate into misleading positioning measurements, particularly when using Wi-Fi signal strength. Similarly, Camp *et al.* [22] provide a detailed explanation of mobility models used on *ad hoc* network research, as well as an evaluation of their performance, and Stoffers *et al*. [23] provide a comparison of radio propagation models.

Oliver *et al*. [24] present a study aimed at exploring users’ physical activity intensity, GPS speeds, and routes traveled during their activities by combining GPS, Geographic Information Systems (GIS), and accelerometry. Their work is relevant for our research due to their analysis of loss of GPS signals due to a range of methodological issues, such as low battery life, signal drop out, and participant noncompliance.

Although these proposals address the positioning problem through collaboration, most of them focus on using a single positioning strategy, and do not take advantage of contextual elements present in the devices’ environment. Moreover, since disaster scenarios have limited or damaged power and communication infrastructure, conventional positioning methods that rely on elements embedded in the environment could operate improperly, resulting in inaccurate measurements, longer waiting times, and unnecessary transmissions that overload the communication link. Summarizing, none of these positioning strategies is able to work properly by considering the main restrictions imposed by the disaster relief scenarios. In order to address this challenge, the next section describes the proposed positioning model.

## 3. Context-Aware Positioning Model

This context-aware positioning model was designed to (a) allow users (*i.e.*, stationary and mobile devices) to estimate their positions in outdoor environments, specifically in urban areas affected by disasters, either by accessing positioning strategies directly, or by sharing positioning information with neighboring users having known positions; and (b) allow a smooth transition between the use of different positioning strategies. Given the model is able to use several strategies for providing this positioning, the process of choosing a particular strategy for a specific node also considers its energy consumption and the impact that it has on the whole positioning process.

A device making use of the model would utilize its peripherals to sense its environment and gather contextual information, which is used to determine a set of suitable positioning strategies from those available in the scenario. Devices with positioning capabilities could either directly access positioning strategies available in the scenario (*i.e.*, self-positioning), such as GPS or radio frequency identification (RFID), or use their neighbors’ positions as reference points (*i.e.*, collaborative positioning).

Similarly, devices that have no positioning capabilities or that are somehow impeded from performing self-positioning could also take advantage of collaboration to estimate their position, using the positioning information of neighbors with known positions as input. Thus, the model could provide this capability to devices with access to at least one positioning strategy present in a given environment, enabling users to perform positioning in situations where they would normally be unable to, as well as favoring positioning strategies that would expend less energy than using the default strategy (usually GPS).

It is important to mention that the model does not aim to improve the accuracy of the devices’ positioning estimations. The model addresses the problem of providing context-aware positioning, with reasonable energy consumption, to devices participating in disaster relief efforts, regardless of whether these devices have the peripherals required to support such activity.

Although the model does not particularly address indoor environments, in several cases these scenarios are also supported, because the positioning is performed considering the nodes with high confidence about their position. However, the focus of the proposed model is on outdoor environments, and therefore the estimations in indoor environments have a low accuracy (but are useful to support several disaster relief activities).

**Figure 1:**
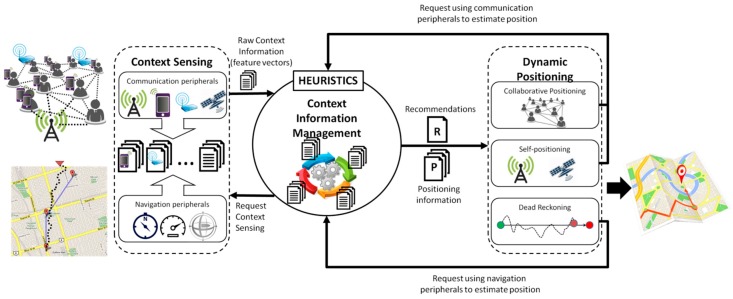
Three stage context-aware positioning model.

Given the nature of the process to be supported, the outdoor positioning is always mandatory and indoor positioning is (in the best case) desirable. Typically, indoor positioning in disaster relief efforts is used only to provide coarse-grained awareness information about the resources’ location (*i.e.*, first responders and equipment), given that the reference points required for such positioning strategies are usually unavailable or in a new location (e.g., in the case of collapsed buildings). Therefore, if the outdoor positioning mechanism used by the first responders is able to provide coarse-grained positioning information when these people are under a roof, the use of a specific indoor positioning strategy is usually discarded. Provided that the proposed model has such a capability, improving its support for indoor positioning is only desirable. This model involves a three-stage process (Figure 1): (1) sensing the context, (2) managing the context information, and (3) performing the context-aware positioning. The following subsections describe the processes considered in these stages.

### 3.1. Context Sensing

**Figure 2:**
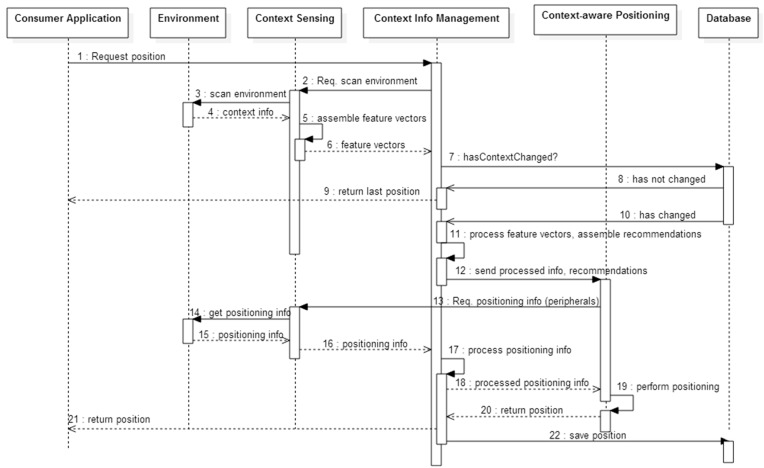
UML sequence diagram of the model’s behavior.

This stage involves sensing the physical environment of a device in order to determine which contextual elements are present at the time a positioning request is sent, as well as interacting with them (Figure 2). To this end, a device must be able to identify and characterize the contextual elements from both its physical environment and other participating devices. These elements are then used to describe and infer the users’ location context.

The contextual elements considered by the model include communication protocols and technologies, positioning strategies, neighboring access points, and neighboring devices. The protocols taken into account are TCP/IP and UDP (transmission control protocol/internet protocol and user datagram protocol, respectively). The technologies are GRPS (general packet radio service), HSDPA (high speed downlink packet access), LTE (long term evolution), Wi-Fi, and Bluetooth.

This model assumes the existence of an *ad hoc* communication infrastructure in the work scenario (in our case, in the affected area) that implements unstable communication links among the nodes, e.g., a mobile *ad hoc* network. The model also considers that the networking issues (like nodes discovery and management of connections/disconnections) are addressed by the communication protocol that implements these links; for instance, the Optimized Link State Routing Protocol (OLSR) [25] or the High Level MANET Protocol (HLMP) [26]. In other words, the positioning model runs into the application layer, and it uses the communication services provided by the networking layer.

As for positioning strategies, several types are considered, including but not limited to fingerprinting, RFID, triangulation, and GPS. Note that the available positioning strategies of a device depend entirely on the device’s capabilities; *i.e.*, if no GPS transceiver is present, the model will not take GPS into consideration, even when it would be the preferred strategy otherwise.

The devices’ peripherals are also considered, since they determine which contextual elements can be sensed and accessed. When the model requests an interaction with the environment, the associated peripherals convey the required data. For example, if the model requests performing dead reckoning, the digital compass will return the device’s current direction and the accelerometer the movement speed.

During the context sensing stage, a device can perform the following tasks: (1) characterizing the physical environment and (2) interacting with that environment. These tasks are described in more detail in the following subsections.

#### 3.1.1. Characterizing the Physical Environment

In order to allow the model to access and consume context information, the latter must be represented in a simple and discrete manner. Thus, the contextual elements are assembled into two feature vectors; one to describe the devices, and the other to describe the physical scenario.

**Table 1:** Devices feature vector.

**Communication Protocols**	List of sensed communication protocols (*i.e.*, TCP/IP and UDP).
**Communication Technologies**	List of sensed technologies (e.g., GRPS, Wi-Fi, *etc*.).
**Positioning Capabilities**	List of sensed positioning strategies (e.g., GPS, fingerprinting, *etc*.).
**Peripherals**	A list of available sensors and actuators, each with their current measurements (e.g., GPS transceiver, Bluetooth, accelerometer, *etc*.)
**Position**	A list of the devices’ last known positions, including their accuracy and positioning decay.
**Fitness**	An indicator of whether the device is apt to participate in (collaborative) positioning.
**Energy**	The device’s maximum and current energy levels.

The devices feature vector (Table 1) stores contextual information based on available peripherals and capabilities. It allows for describing devices in terms of both their capability to sense and access the environment and their own fitness to participate in the positioning process. This means a device could behave differently depending on which contextual elements are available at a given time.

The fitness score is a dynamic indicator of a device’s aptitude to perform positioning. The greater the fitness of a device, the more likely its positioning information could be used during collaborative positioning. Formally, fitness is calculated as follows:

(1) $$\text{F}=\text{D *}\left(\text{A}\frac{{\text{A}}_{\text{i}}-1}{{\text{A}}_{\text{i}}}\right)\text{* P *}\frac{\text{E}}{{\text{E}}_{\text{max}}}$$

Where F is the fitness score of a device, which can range from 0 to 100. D  is the decay of a positioning estimation. A is the accuracy requirement of the consumer application, and ${\text{A}}_{\text{i}}$ the accuracy of the last positioning estimation (both percentages). P is a binary value representing whether the device knows its position or not. E is the current amount of energy, and $E_{max}$ the maximum energy level of the device, both represented as arbitrary units.

Devices with greater decay (*i.e.*, lower D values), lower accuracy, and/or lower energy values would tend to have low fitness scores. On the other hand, devices with lower decay (*i.e.*, greater D values), greater accuracy, and/or energy values would yield higher fitness scores. Devices that do not know their position would have a fitness of zero (if P=0, then F=0), since they do not contribute to the positioning process.

The scenario feature vector requires describing the contextual elements in the devices’ proximity. For the purposes of our work, this characterization actually includes both the representation of the direct context of a device and the positioning strategies that can be accessed by such a device, as shown in Table 2.

**Table 2:** Scenario feature vector.

**Access Points**	List of access points available in a device’s context.
**Neighbors**	List of neighboring nodes available in a device’s context.
**Positioning Strategies**	List of positioning strategies available in a device’s context.

Thus, a device would sense and update its scenario feature vector each time it attempts to perform positioning, effectively assembling a map of its surrounding area (*i.e.*, represented through the device’s context). Once a device has scanned its environment, it will build a feature vector for each neighboring device within communication range, storing them as an Extensible Markup Language (XML) file. We decided to use an XML representation not only because it is a standard format, but also because it is flexible and allows for information integration using well-known mechanisms. Thus, several feature vectors can be integrated into a large one, involving low computing effort.

Given the diversity of organizations participating in disaster relief efforts, counting on a common representation of the shared information is mandatory to ensure data interoperability. In this sense, the use of XML to represent the shared information seems to be the best option.

Note that for purposes of the model, access points are treated as stationary devices, and therefore each has its own feature vector. Once this task is completed, the XML file is sent to the next stage, *i.e.*, the management of contextual information.

#### 3.1.2. Interacting with the Physical Environment

This task deals with the use of a device’s communication and navigation peripherals to interact with the environment. It is usually requested during the context-aware positioning stage, as a means to access positioning strategies or collaborate with neighboring devices.

Communication peripherals include all types of peripherals used to send or receive information, such as Global System for Mobile Communications (GSM) and Wi-Fi antennas, RFID card readers, or GPS transceivers. Navigation peripherals include the magnetometer or digital compass, accelerometer, and gyroscope. These peripherals are used to determine the general direction and speed of a device, allowing for a degree of relative positioning (dead reckoning). The information gathered during this task is assembled as a file and sent to the management of contextual information stage for its processing.

### 3.2. Management of Contextual Information

This second stage is key for the model. In this phase the contextual elements are assessed and processed in order to eliminate redundant and irrelevant information related to the positioning of a device. Based on this processed information, a set of recommendations is assembled. These recommendations contain the positioning strategies that can be accessed by the device, prioritized based on the device’s current context and expected energy consumption level. The reference values for this last feature were obtained from several studies reported in the literature.

Three tasks can be performed during this stage: (1) request sensing the physical environment; (2) process context sensing feature vectors and assemble recommendations; and (3) manage positioning information. Next we describe these tasks.

#### 3.2.1. Request Sensing the Physical Environment

This task is triggered when a consumer application requests the model to perform positioning. The contextual information manager sends a call for scanning the environment towards the context sensing stage (Figure 2). The goal is to obtain the current characterization of the physical environment.

The result is a raw XML file containing feature vectors describing the scenario, and each surrounding device, which is sent to the following task for processing. This file also contains data read from peripherals, if applicable.

#### 3.2.2. Process Context Sensing Feature Vectors and Assemble Recommendations

During this task, the scenario feature vector is processed, extracting relevant, ignoring irrelevant, and updating redundant information by means of low-level data fusion [27]. The model uses the raw contextual information obtained during the context sensing stage, along with any other data stored from previous positioning attempts, in order to produce an up-to-date representation of the scenario.

The first step is to prune neighbors that are not useful for the positioning process. To this end, all nodes with fitness scores below a threshold of 60 (on a scale of 1–100) are discarded; the remainder are used as input. This threshold is dynamic, and can be lowered by the model in subsequent positioning attempts if the number of nodes remaining is too low to perform positioning. For example, if no nodes are found at a fitness threshold of 60, the next positioning attempt will be performed at 55; if only one node is found, the next attempt will be at a threshold of 50, and so on. Conversely, if the average fitness of the pruned nodes is over the current threshold, the threshold for the next positioning attempt will increase accordingly. If the threshold ever decreases to 20, the node’s position is automatically set to unknown, and the threshold is reset to its initial value of 60.

The model must then compare and update information from the contextual and stored data. The structure of the XML feature vectors allows for rapid comparison of discrepancies between a node’s current and previous scenario information, facilitating this process. If a discrepancy is found, it is immediately updated. For example, if we have information from node $N_{7}$ in storage, and receive new information from context sensing $N_{7}^{'}$), the model will probably detect changes only in the positioning capabilities, energy, and position features, since they change over time.

On the other hand, communication protocols, communication technologies, and peripherals features are likely to remain the same unless direct action from the user is taken to shut them off. If changes are detected, the data is updated.

The remaining nodes are then ordered based on their fitness scores. The resulting subset should contain only viable candidate nodes for collaboration, essentially a summary of the work context of a device. This summary is in fact a trimmed version of the original scenario feature vector, which only contains the positioning information of viable neighbors, as well as the positioning strategies available in the target device’s current context.

The next step involves assembling the recommendations using the information from the summary. To this end, the positioning strategies are treated as classes and represented as a vector (Table 3). Each strategy in the vector has an associated score, which represents the likelihood of a device belonging to a class (*i.e.*, positioning strategy) given its contextual information. The higher this score, the greater the estimated suitability of the associated positioning strategy. All scores must add up to 1.0, regardless of the number of positioning strategies observed by the model.

**Table 3:** Recommendation vector example.

Strategies	S1	S2	S3	S4	S5	C1	C2	C3
**Score**	0.35	0.15	0.05	0.10	0.30	0.0	0.00	0.05

A total of eight positioning strategies have been considered in our model, although additional strategies can be added if required. Five of these strategies are dependent on the scenario and the devices’ positioning capabilities: GPS, A-GPS, Cell-ID, fingerprinting, and RFID (S1 through S5). The other three can be estimated directly by the model by using information from the summary: triangulation, minimum bounding rectangle, and dead reckoning (C1, C2, and C3, respectively).

In order to build the set of positioning recommendations of a device, the model attempts to determine the suitability of the positioning strategies, by using a randomized decision tree. Each time the model attempts to classify a device, it picks a “remaining” attribute from the device’s associated scenario feature vector at each node expansion. A categorical feature (such as positioning capabilities) is considered “remaining” if the same categorical feature has not been chosen previously in a particular decision path starting from the root of tree to the current node. However, continuous features (such as fitness or current energy level) can be chosen more than once in the same decision path, and each time that such a feature is chosen, the threshold is determined randomly.

Depending on which decision path is chosen in each decision node, the scores of the recommendation vector’s positioning strategies are modified. Once all the attributes from the device’s feature vector for such a scenario have been chosen (*i.e.*, there are no remaining features), the recommendation vector is considered to be assembled.

Table 4 shows an example of the training data used to tailor the thresholds of the decision tree’s nodes. Each training scenario feature vector (representing the context of a device) carries an associated recommendation vector, with empirically determined scores obtained from controlled real world experiments, and from literature. This data was used to determine the values of the thresholds for each of the feature vector’s attributes, and their effect over the recommendation vector’s strategy scores.

**Table 4:** Training data example.

	S1	S2	S3	S4	S5	C1	C2	C3
**Scenario Feature Vector 1**	0.35	0.15	0.05	0.10	0.30	0.0	0.0	0.05
**Scenario Feature Vector 2**	0.0	0.0	0.0	0.0	0.0	0.0	0.0	1.00
**Scenario Feature Vector 3**	0.80	0.0	0.0	0.0	0.0	0.0	0.0	0.20
···	···							

**Figure 3:**
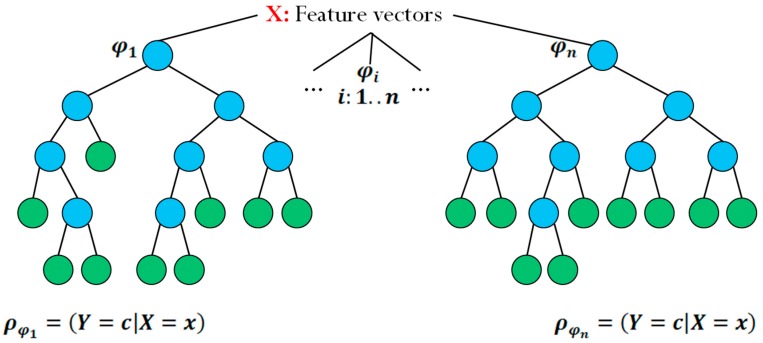
Example of randomized decision trees.

Figure 3 shows an example of two randomized trees. X represents the input of the tree, the device’s associated scenario feature vector. $\varphi_{1}$ and $\varphi_{n}$ represent two different random decision trees, and $\varphi_{i}=\left(Y=c\text{|}X=x\right)$ represents the probability of obtaining the recommendation set Y given feature vector x.

Once the summary and recommendation vectors have been assembled, they are stored locally for future reference. The next time that a positioning request is sent to the model, the context information manager will assemble a new summary and compare it with the latest it has stored, in order to check for changes in the navigation context of the device. If the model determines that the navigation context has not changed (*i.e.*, the user has not moved), the manager skips the context-aware positioning stage and just sends back the last estimated position (if available) to the consumer application. Otherwise, the positioning process is performed as usual. Both the contextual information summary and the set of recommendations are the inputs used during the context-aware positioning.

#### 3.2.3. Manage Positioning Information

This task is conducted just after the context sensing has determined which positioning strategy will be used, based on the set of recommendations. If the recommendation is to perform self-positioning, then the context information manager would receive a request to access the corresponding peripherals (e.g., a GPS transceiver) in order to determine the position of the device. Note that the model only determines which strategy is to be used, and has no control on whether the peripherals manage to obtain the required position or not; this is entirely dependent on the device’s capabilities. If all goes well, the position is obtained and processed. In the event of a time-out (*i.e.*, no positioning estimation could be obtained), a new request is sent by the model to the peripherals related to the next available strategy from the recommendations vector, and so on.

On the other hand, if the recommendation is to perform collaborative positioning, the model would request the context-aware positioning component to perform positioning based on that recommendation. Unlike self-positioning, this process is entirely dependent on the model, and no peripherals are required since all the required information is already in the summary. Either way, once the position of the device has been determined, it is stored for future reference, and redirected towards the consumer application.

### 3.3. Context-Aware Positioning

The third stage of the model performs the positioning estimation, which is conducted based on the recommendations assembled during the management of contextual information stage. When required, the model will attempt to perform positioning using the recommended candidate strategies, according to the contextual information and priorities included in the summary. Two tasks are performed during this stage: (1) request self-positioning, and (2) perform collaborative positioning. These tasks are explained in the next subsections.

#### 3.3.1. Request Self-Positioning

If a device using the model has positioning capabilities of its own, it is very likely that the recommendation for positioning will be to perform self-positioning, i.e., accessing a strategy available in the scenario itself. In that case, the model would send a request to use the respective peripherals, and estimate its position on its own. For example, devices with GPS transceivers or RFID readers could activate them, while Android-based smartphones could use their embedded Wi-Fi positioning system in order to estimate their positions.

**Figure 4:**
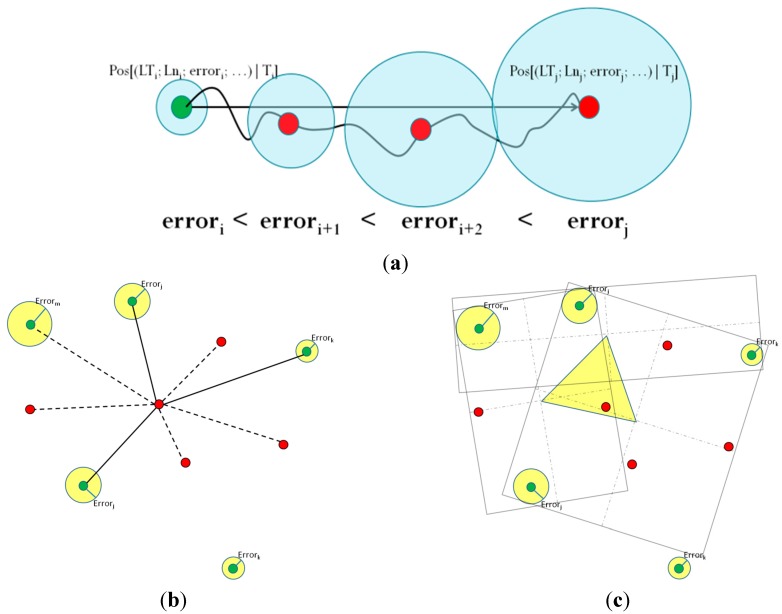
(**a**) Dead reckoning; (**b**) trilateration; (**c**) minimum bounding rectangle (MBR).

Dead reckoning (Figure 4a), also known as deduced reckoning or DR, a special type of self-positioning, is the process of calculating one’s current position by using a previously determined position, or fixing and advancing that position based upon known or estimated speeds, over elapsed time and course [28]. In the context of our model, dead reckoning is applied using the list of known positions, in conjunction with the navigation peripherals, in order to smooth transitions between different positioning strategies, since there could be a “blind” positioning elapsed time between using different strategies, or between two consecutive estimations. However, DR is subject to cumulative errors, and it requires means to correct its estimations by using a proper positioning strategy [28]. It is by no means reliable for long periods, and is used only to offer positioning consistency during “blind gaps” when the device does not have access to a proper positioning strategy. The proposed model strictly ignores all nodes using dead reckoning during the collaborative positioning process.

It is important to note that the actual self-positioning conducted by a device is transparent to our model, *i.e.*, the model recommends using a positioning strategy, but it is up to the device’s peripherals to actually perform the position estimation.

#### 3.3.2. Request Collaborative Positioning

If the recommendation is to perform collaborative positioning, the model will use the contextual information of the summary to select viable neighboring devices with known positions as reference points. Then, it proceeds to estimate the device’s position using either tri-lateration, or minimum bounding box approximation (MBB).

Lateration is a type of triangulation in which the position of a resource is estimated by measuring its distance to several reference points with known positions. Using the direction or length of the vector drawn between the location to be estimated and the reference points, it is possible to calculate the absolute position of the desired resource [29]. Figure 4b shows an example of tri-lateration under the model. The green nodes know their positions with fitness scores over the threshold of 60; red nodes are those that do not know their positions, or that have a low fitness score and therefore cannot collaborate with the target. The solid lines link the target to the nodes chosen as reference points, while the dotted lines link nodes that have been acknowledged but are not part of the collaboration process.

The model requires at least three reference points to perform tri-lateration, although more points could be used to improve the accuracy of the estimation, if available (multi-lateration). An advantage of this method is that it involves a small setup effort [30].

The smallest or minimum bounding box is, for a point set (S) in N dimensions, the box with the smallest measure (area, volume, or hypervolume in higher dimensions) within which all specified points of S lay. The MBB of a point set is the same as the MBB of its convex hull, a fact that may be used heuristically to speed up computation [31]. The term “box” comes from its usage in the Cartesian coordinate system, where it is indeed visualized as a rectangle in two dimensions (2D), a rectangular parallelepiped in three dimensions (3D), *etc*. Figure 4c illustrates an example of MBB applied to 2D positioning. Using as many nodes that know their positions as possible, a series of rectangles is formed, each containing at least three nodes. Then, line segments are traced from the centers of these rectangles to each other, forming an irregular polygon (in this case, a triangle), which represents the area where the node is likely located. In some cases, this polygon provides better accuracy than using tri-lateration. However, the cost of calculating this polygon increases in direct relation with the number of rectangles formed.

## 4. Experimental Design

The purpose of this section is to provide technical information on the inner workings of the simulation scenario, and it is intended for fellow researchers who would want to replicate our experiment. If the focus of the reader is only on the model, this section can be skipped safely.

The experiments were conducted using a series of outdoor simulated scenarios, each populated with three types of nodes: stationary nodes, pedestrians, and vehicles. Some of these nodes have positioning capabilities (e.g., a computing device with GPS), and they interact with each other through a mobile *ad hoc* network (MANET) to sense and share contextual elements.

All scenarios were designed using the Network Simulator 3 (ns-3), a networking simulator built as a core system with a set of interchangeable libraries that can be linked or imported to user programs (simulations). It provides substantial support for simulation of networking scenarios and is highly scalable, extensible, and modular [32]. The versatility of the ns-3 allows users to write simulation scenarios (based on already validated models) using only the features they want to represent, as well as adding their own libraries to the core of the simulator.

The nodes mobility was modeled using the BonnMotion simulator [33], since this tool includes well-known models that represent people mobility in the study scenarios, particularly for disaster areas [34]. The mobility models considered in this work have a widely analyzed and validated mathematical basis [22,35,36].

The following subsection describes the parameters of the simulated environment and the conditions under which the simulations are performed, as well as the implementation of the experimental test bed.

### 4.1. Experiment Description

Our simulation scenario uses the IEEE 802.11b standard for Wireless Local Area Networks, and considers mobile nodes forming a MANET. The simulated network allows for retransmission of lost packets due to collision or propagation loss [37]. The chosen propagation loss model is the maximal range, in which the propagation loss is determined based on the distance between transmitter and receiver (*i.e.*, the communication threshold). Any transmission within this threshold is received at maximum transmission power, while transmissions beyond that threshold are received at −1000 dBm (effectively zero), and are therefore dropped. The simulations observe thresholds of 30, 50, and 80 m. In order to allow for more variation, we also use the random propagation delay model, using a time threshold of 0.7 s. If the random delay is over that threshold, the packet is dropped by the receiver. To avoid flooding the network with requests, we only allow communication between nodes at a distance of one hop.

Regarding the nodes, pedestrians represent people on foot, with a random direction and speed in the ranges of [0, 2π] and [1.0, 4.0] m/s, respectively, and movement based on the random walk mobility model [33]. Vehicle nodes represent people riding bicycles, cars, motorbikes, or similar land transportation vehicles. These nodes use the random waypoint mobility model [33], and have a speed in the range of [5.0, 20.0] m/s. Both vehicle and pedestrian nodes move for a time t or a distance d, and then choose a new random direction and speed. Stationary nodes, on the other hand, represent devices that are immobile or doing micro-mobility, such as parked vehicles, people sitting around, or working in newsstands or hotdog carts. These nodes use the constant position mobility model, in which they have an effective speed equal to zero, although their direction can still change. Nodes that begin the experiment as stationary never move during the simulation process, but pedestrians and vehicles can randomly become stationary for certain time periods.

Each node has a role, depending on its positioning capabilities. They can either be beacons (also known as anchors), or non-beacons. Beacon nodes have access to at least one positioning strategy (e.g., GPS). Non-beacon nodes, on the other hand, have no positioning capabilities whatsoever.

The simulation scenario consists of an area of 500 × 500 m^2^, representing a park or a university campus, and is populated by the three types of nodes, in the following proportions: 65% pedestrians, 25% vehicles, and 10% stationary. All nodes are randomly positioned within the simulated scenario, and each node has a random initial direction and speed. The number of nodes we have considered is 100 for each simulated scenario.

As for the roles, three different configurations of beacons can be assigned to a given scenario: 10%, 25%, and 50% of (beacon) nodes, respectively. In order to observe the impact of the nodes’ speed during the positioning process, we first assigned the beacon role randomly to any type of node, then only to pedestrian nodes, and finally only to vehicles. Since we have only 100 nodes for each scenario, we had to increase the number of vehicles for the third scenario, reducing the other types of nodes proportionally in order to ensure that the results are not biased. All nodes begin with 100% energy, and they do not know their position at the beginning of the simulation.

We assume that all participating devices are using the proposed context-aware positioning model. This means all nodes can potentially sense their environment and collaborate with their neighbors. The communication and navigation capabilities, represented in the simulation by the devices feature vector, are assigned randomly to all nodes. Thus, node A could have a feature vector showing that it possesses all communication protocols, three out of five communication technologies, no positioning capabilities, one navigation peripheral, *etc*. (see Equation (2)):

(2) [[1 1][1 0 1 1 0][0 0 0…][[1 0 0]…]…]

Meanwhile, node B could have only UDP, two communication technologies, GPS, two navigation peripherals, *etc.* (Equation (3)):

(3) [[0 1][0 0 0 1 1][1 0 0…][[0 1 1]…]…]

This allows for a wide range of device configurations, and provides device heterogeneity to the simulations.

Since the scenario is highly volatile (*i.e.*, most nodes are moving), we have assigned a decay indicator to the positioning estimations, in the range of 0 to 100, representing the “validity” of these estimations as time passes and the nodes move. The decay is represented by the following formula:

(4) $$\text{D}\left(\text{s},\text{t}\right)=100-\text{k *}\overset{\text{t}}{\underset{\text{i}=1}{\sum }}{\text{s}}_{\text{i}}$$

Where D is the decay, s is the speed of the node, t represents the time from the last positioning estimation, and k is a factor determined based on the speed of the node.

Basically, the decay indicator begins with a value of 100. Then, it decreases based on the speed of a node over the course of a simulation. Once the decay reaches a threshold of 60, the model will request a new positioning estimation. If the decay reaches a threshold of 20, the node’s position is set to unknown.

At the beginning of a simulation, all nodes start sensing their context, attempting to estimate their position. Only beacon nodes are able to position themselves at first, but eventually non-beacons obtain positioning information and thus are able to estimate their positions. If a node is unable to obtain positioning information, its last known position (if available) will continue decaying until it either manages to estimate its position, or the decay hits a threshold of 20.

We let the nodes interact for 30 s, in order to allow the positioning decay to converge, and then we begin tracing four features: (1) the decay of non-beacon nodes’ positions; (2) the number of non-beacon nodes that know their positions with decay of at most 60; (3) the number of non-beacon nodes that know their positions with any degree of decay; and (4) the energy consumption of all nodes. The simulations last for 300 s after the convergence time.

### 4.2. Experiment Implementation

The steps followed in order to build an experiment based on the ns-3 script mockup are detailed below. First we explain how we can represent the model in the simulation scenario using an application class; then we describe the behavior of the nodes and the mobility models that can be used; and finally we show how to set up channels for communication between nodes.

#### 4.2.1. Application Setup

The ns-3 allows us to easily set up the behavior of the model. Algorithm 1 shows a general example of an ns-3 application class, commApp, which can be attached to the nodes. This application allows for configuring the nodes’ behavior under certain circumstances, such as performing an action after moving a specific distance, what to do after receiving a given message, and so on.

Please note that commApp is just an outline of the actual class used during our experiments, which includes far more functionality. Since the ns-3 does not support positioning per se, the commApp class is used to emulate a positioning application, represented by the UpdatePositionDecay and UpdatePosition methods. UpdatePositionDecay is in charge of calculating and updating the decay of a positioning measurement, and UpdatePosition is invoked to attempt to perform positioning once the decay has reached the model’s positioning error threshold.

Using this class allows us to determine which sockets will be used for communication, the number of packets that will be transmitted, their size, and the interval at which they are sent. It also allows for setting up triggers when sending or receiving messages, as well as determining the conditions upon which it will begin or stop making requests. Additional functions and variables can be added or modified depending on what we want to achieve with the simulation. Any number of applications can be attached to a node, as required.

**Algorithm 1** Application Class Example
1.	class commApp:public Application{
2.	 public:
3.	  commApp ();
4.	  virtual ~commApp();
5.	  void Setup (Ptr<Socket> socket,
           uint32_t packetSize,
           uint32_t nPackets,
           Time pktInterval);
6.	 private:
7.	  virtual void Start (void);
8.	  virtual void Stop (void);
9.	  void ScheduleTx (void);
10.	  void SendPacket (void);
11.	  void UpdatePositionDecay(void);
12.	  Void UpdatePosition (void);
13.	  Ptr<Socket> m_socket;
14.	  uint32_t    m_packetSize;
15.	  uint32_t    m_nPackets;
16.	  Time        m_pktInterval;
17.	  EventId     m_sendEvent;
18.	  bool        m_running;
19.	  uint32_t    m_packetsSent;
20.	  uint32_t    m_decayRate;
21.	  uint32_t    m_decayThreshold;};


#### 4.2.2. Nodes and Mobility

The ns-3 provides helper classes, which work like interfaces and simplify the overall configuration process of a simulation. Algorithm 2 shows the NodeContainer helper class, which allows us to create and store groups of nodes that can be batch-configured. Instead of configuring each node individually, configurations can be applied to a container and all nodes within will receive that configuration.

Once the nodes are created, they must be assigned a position within the scenario, which can be done using the MobilityHelper class. Algorithm 3 shows examples of two position allocators, Grid and RandomRectangle. The Grid allocator places the nodes in a grid in which the center coincides with the center of the scenario, separated by DeltaX and DeltaY units from each other. The RandomRectangle creates a bounded area within which the nodes are randomly positioned. Other position allocators such as List allow the user to define a list of positions, and let the simulator pick the positions sequentially for each node in order. During our experiments, we have made extensive use of the RandomRectangle and List position allocators.

**Algorithm 2** Node Creation
1. NodeContainer walkerNodes;
2. walkerNodes.Create (65);
3. NodeContainer vehicleNodes;
4. vehicleNodes.Create (25);



**Algorithm 3.** Position allocation
1. MobilityHelper mobility;
   //Grid
2. mobility.SetPositionAllocator(
    "ns3::GridPositionAllocator",
    "MinX", DoubleValue (0.0),
    "MinY", DoubleValue (0.0),
    "DeltaX", DoubleValue (distance),
    "DeltaY", DoubleValue (distance),
    "GridWidth", UintegerValue (10),
    "LayoutType",StringValue("RowFirst")
    );
  //Random Rectangle
3. mobility.SetPositionAllocator("ns3::
RandomRectanglePositionAllocator", "X",StringValue("ns3::UniformRandomVariable[Min=-250.0|Max=250.0]"), "Y",StringValue("ns3::UniformRandomVariable[Min=-250.0|Max=250.0]"));



**Algorithm 4** Assigning Mobility
1. MobilityHelper mobility;
 //Constant Position
2. mobility.SetMobilityModel("ns3::ConstantPositionMobilityModel");
 //Random Walk based on time
3. mobility.SetMobilityModel("ns3::RandomWalk2dMobilityModel",
"Bounds", RectangleValue(Rectangle (-250, 250, -250, 250)),
"Speed",StringValue("ns3::UniformRandomVariable[Min=6.0|Max=20.0]"),
  "Time", TimeValue(Seconds(1)),
  "Mode", StringValue ("Time"));
//Random Walk based on time
4. mobility.SetMobilityModel("ns3::RandomWalk2dMobilityModel",
  "Bounds",RectangleValue(Rectangle(-250, 250,-250, 250)), 
  "Distance", DoubleValue(30),
  "Mode", StringValue ("Distance"));
5. mobility.Install (walkerNodes);



Finally, the nodes must be assigned mobility models. The ns-3 has a comprehensive list of these models, including random walk, random waypoint, random direction, Gauss-Markov, etc. Moreover, user-made models can easily be imported into the simulations to fit a specific scenario. Algorithm 4 shows two models, the Constant Position, which is self-explanatory, and the Random Walk 2D based on Time and on Distance. In the Random Walk 2D model based on Time, the nodes move for time t (in seconds) and then randomly change direction and speed. For the Random Walk 2D based on distance, nodes move distance d and then change direction and speed randomly. If we want the nodes to follow a predefined path, we can assign direction, speed, and waypoints manually, and modify these values through triggers at predefined times.

Once the position allocator and mobility models have been set, they must be assigned to a node, or NodeContainer. Only one allocator and mobility model can be assigned to a single node, but any number of nodes can have any combination of allocator and mobility model during a single simulation.

#### 4.2.3. Communication Behavior

Once the nodes and the application have been set up, the scenario must be configured. Algorithm 5 shows an example of how the environmental variables are set up in an ns-3 script. Algorithm 6 shows an example of the configuration of a communication channel, in this case Wi-Fi.

As stated before, the scenario observes the IEEE 802.11b wireless standard with a Request To Send/Clear To Send (RTS/CTS) handshake set for frames above 2200 bytes, disabling fragmentation for frames under that value. Retransmission of lost packets due to collision or propagation loss is allowed [37], which means the nodes will make sure they can establish communication and then use a redundant protocol to make sure they receive packets correctly. The transmitted information consists of a device’s contextual information (*i.e.*, their feature vector), as well as its unique node identifier.

The ns-3 offers several propagation models for signal delay and signal loss, which represent the power fluctuations of the signal under different real-world conditions. Examples are the Constant Speed Propagation Delay model, which has a constant signal speed independent of the distance; the Building Propagation Loss, which simulates shadowing and wall penetration signal loss; or the Okumura Hata, which is used to model path loss in open areas for distances of over 1 km and frequencies ranging from 150 MHz to 2.0 GHz [38]. A propagation delay model can be used together with a loss model, if they are compatible.

**Algorithm 5** Scenario Configuration
1.   std::string phyMode ("DsssRate1Mbps");
2.   double distance = 50; // meters
3.	 uint32_t packetSize = 1000; // bytes
4.	 uint32_t numPackets = 10000;
5.	 double interval = 1.0; // seconds
6.	 Time interPacketInterval = Seconds(interval);
7.	 Config::SetDefault("ns3::WifiRemoteStationManager::FragmentationThreshold", StringValue ("2200"));
8.	 Config::SetDefault("ns3::WifiRemoteStationManager::RtsCtsThreshold", StringValue ("2200"));
9.	 Config::SetDefault("ns3::WifiRemoteStationManager::NonUnicastMode", StringValue (phyMode));



We began our experiments using the Friis propagation model, which gives the power received by one antenna under idealized conditions given another antenna some distance away is transmitting a known amount of power. The model uses the following equation:

(5) $$P_{r}=\frac{P_{t}G_{t}G_{r}\lambda^{2}}{{\left(4\pi d\right)}^{2}L},$$

**Algorithm 6** Scenario Configuration
1.	WifiHelper wifi;
2.	wifi.SetStandard(WIFI_PHY_STANDARD_80211b);
3.	YansWifiPhyHelper wifiPhy = YansWifiPhyHelper::Default ();
4.	wifiPhy.Set("RxGain",DoubleValue(0));
5.	wifiPhy.SetPcapDataLinkType(YansWifiPhyHelper::DLT_IEEE802_11_RADIO); 
6.	YansWifiChannelHelper wifiChannel;
7.	wifiChannel.SetPropagationDelay("ns3::ConstantSpeedPropagationDelayModel");
8.	//wifiChannel.AddPropagationLoss("ns3::FriisPropagationLossModel");
9.	wifiChannel.AddPropagationLoss("ns3::RangePropagationLossModel","MaxRange", DoubleValue (50));
10.	wifiPhy.SetChannel(wifiChann.Create());
11.	NqosWifiMacHelper wifiMac = NqosWifiMacHelper::Default ();
12.	wifi.SetRemoteStationManager ("ns3::ConstantRateWifiManager", 
"DataMode",StringValue (phyMode), "ControlMode",StringValue (phyMode));
13.	wifiMac.SetType ("ns3::AdhocWifiMac");
14.	NetDeviceContainer walkerDevices = wifi.Install (wifiPhy, wifiMac, walkerNodes);
15.	NetDeviceContainer vehicleDevices = wifi.Install (wifiPhy, wifiMac, vehicleNodes);
16.	NetDeviceContainer immobileDevices = wifi.Install(wifiPhy, wifiMac, immobileNodes);
  //Assigning Networks to Nodes
17.	InternetStackHelper internet;
18.	internet.Install (walkerNodes);
19.	internet.Install (vehicleNodes);
20.	internet.Install (immobileNodes);
21.	Ipv4AddressHelper ipv4;
22.	NS_LOG_INFO ("Assign IP Addresses.");
23.	ipv4.SetBase("10.1.1.0,"255.255.255.0);
24.	Ipv4InterfaceContainer iWalk = ipv4.Assign (walkerDevices);
25.	ipv4.SetBase(10.1.2.0",255.255.255.0");
26.	Ipv4InterfaceContainer iVehi = ipv4.Assign (vehicleDevices);
27.	Ipv4InterfaceContainer iImmo = ipv4.Assign (immobileDevices);



Where $P_{r}$ is the reception power; $P_{t}\;$ is the transmission power; $G_{t}$ is the transmission gain; $G_{r}$ is the reception gain; λ is the wavelengt  d; is the distance between transmitter and receiver (m); and L is the system loss. The λ value is commonly calculated as C/f, where C= 299792458 m/s (the speed of light in a vacuum); and f is the frequency (Hz), which can be configured by the user via the Frequency attribute. However, the Friis model is valid only for propagation in free space within the so-called far field region, which can be considered as approximately the region for d>3λ. The model will still return a value for d>3λ, as doing so (rather than triggering a fatal error) is practical for many simulation scenarios. However, we stress that the values obtained in such conditions cannot be considered realistic [39].

Several other propagation models were tested, until finally settling on using a modified version of the maximal range model with a communication threshold of 50 m, representing the maximum communication range of the participant nodes. This propagation model is used in conjunction with a modified random propagation delay model, with a 0.7-second listening threshold to drop packages.

Algorithm 6 shows an example of the Wi-Fi channel configuration. Once configured, the network layer must be assigned to the nodes. The InternetStackHelper provides the nodes with IP/TCP/UDP functionality, allowing setting communication capabilities with ease. Note that the model supports different communication channels; however, due to ns-3 limitations, we have focused on using Wi-Fi as the channel of choice, and simulated different channels using different networks. The nodes can either use the same network, or separate networks with common nodes working as links (thus emulating the use of different channels), with a one-hop communication restriction.

**Algorithm 7** Source and Sink Examples
				  
1.	TypeId tid = TypeId::LookupByName ("ns3::UdpSocketFactory");
//Assigning Sinks to walker nodes
2.	Ptr<Socket> recvSink;
3.	for(uint32_t iNode=0; iNode<65;  iNode++){ 
    recvSink = Socket::CreateSocket (walkerNodes.Get (iNode), tid);
    local = InetSocketAddress (Ipv4Address::GetAny (), 80);
    recvSink->Bind (local);
    recvSink->SetRecvCallback(MakeCallback (&ReceivePacket));}
//Assigning Source to node 0
4.	Ptr<Socket> source;
5.	source = Socket::CreateSocket (walkerNodes.Get (0), tid);
6.	InetSocketAddress remote = InetSocketAddress(Ipv4Address ("255.255.255.255"),80);
7.	source->Connect (remote);
//Adding the app to the source
8.	Ptr<MyApp> app = CreateObject<MyApp> ();
9.	app->Setup(source,1040,10000, Seconds(1.));
10.	walkerNodes.Get(0)->AddApplication (app);
//Scheduling the transmission
11.	app->SetStartTime (Seconds (1.));
12.	app->SetStopTime (Seconds (20.));




With this, the experiment observes the overall network structure of a mobile ad-hoc network (MANET); each node has IP/TCP/UDP, IPv4, and routing capabilities; and each group of nodes within range will form their own network, trading positioning information with each other.

In order to allow the nodes to communicate, they must be assigned the roles of transmission sources and sinks (Algorithm 7). Sources are nodes with transmission capabilities, while sinks are nodes listening on a particular port. For the purposes of our experiment, all nodes are both source and sink, since they must collaborate with each other. To this end, each node requires two pairs of sockets to be created: one for sending handshake requests and another to listen for answers; and two more for the actual response of the neighbors. Each pair of sockets is linked to a certain port number, so that collisions can be avoided when receiving multiple positioning requests and collaboration responses.

Finally, the commApp application can be attached to source nodes, as shown in Algorithm 7. By attaching the application, the behavior of the nodes during the simulations can be configured as required. With this, the simulation is ready to be executed. The next section shows the results obtained after running the battery of simulations.

## 5. Analysis of Results

This section shows the results obtained from our experiments. First we show the exploratory results of the simulations of an early version of the model; then we explain our findings; and finally we show results obtained from a simulation using an improved version of the model.

Results from the initial experiments have not been included in this work, since those were used to build and improve the classification tree, as well as determining the initial fitness thresholds of the model.

### 5.1. Exploratory Results

This subsection shows results obtained from the battery of simulations described in section 4. These results were analyzed and used to improve the model. For purposes of a better understanding of the presented results, we will refer to the average fitness as “confidence”, representing the likelihood that a device can estimate its position if it performs positioning collaboratively. This is done to reflect the fact that higher average fitness levels imply a higher confidence in obtaining a successful positioning estimation.

Three variables have been monitored during our experiments: the confidence level of the scenario, the average number of nodes over given thresholds; and the global energy consumption of the scenario. The objective of these experiments was to determine how the communication range of the nodes and the number of beacons present in a scenario affected the observed variables.

**Figure 5:**
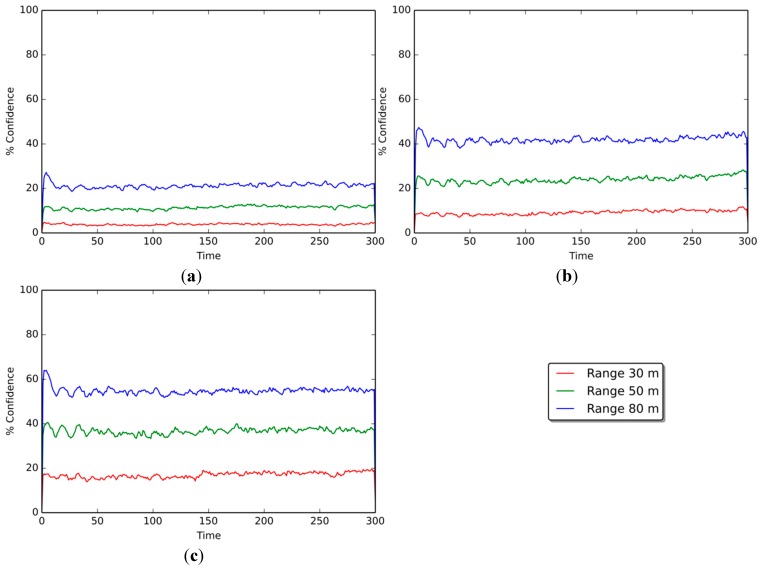
Average confidence of non-beacon nodes per range, in scenarios with (**a**) 10 beacon nodes, (**b**) 25 beacons, and (**c**) 50 beacons.

Figure 5 shows the confidence levels of the positioning estimations of non-beacon nodes only. Results are shown for scenarios with 10%, 25%, and 50% beacon nodes; the curves on each graph represent the confidence level at communication ranges of 30, 50, and 80 m, respectively.

Figure 5a illustrates results for the 10% beacon scenario. The confidence level reaches averages of 4.05% at a communication range of 30 m, 11.26% at a range of 50 m, and 22.07% at 80 m. The 25% beacon scenario (Figure 5b) shows slightly better results, reaching average values of 9.46%, 24.32%, and 41.89% for each of the communication ranges. As for the 50% beacon scenario (Figure 5c), the confidence levels reached 16.67%, 36.48%, and 54.95% for the 30-, 50-, and 80-m communication ranges, respectively.

Results show that the greater the communication ranges of the devices, the greater the confidence levels of the scenarios, as expected. The increment in the confidence level in Figure 5a (10% beacon scenario) is +7.21% when augmenting the range from 30 to 50 m, and +10.81% when going from 50 to 80 m. In a similar manner, the confidence level grows consistently as the number of beacons present in the scenario increases, amounting to +14.86% and +17.57% in Figure 5b (25% beacon scenario), and +19.82% and +18.47% in Figure 5c (50% beacon scenario).

If we look at the behavior of each curve across the three confidence scenarios, as more beacon nodes are available, the confidence level also rises. For the 30-m communication range, when going from 10% beacons to 25% beacons (Figure 5a,b, respectively), there is an increase of 5.40% in the confidence level, and of 7.21% when going from 25% beacons to 50% (Figure 5b,c). For the 50-m communication range, the increase in confidence is 13.06% and 12.16% when going from 10% to 25% beacons and from 25% to 50%, respectively. As for the 80-m communication range, the increase amounts to 19.82% and 13.06%.

**Figure 6:**
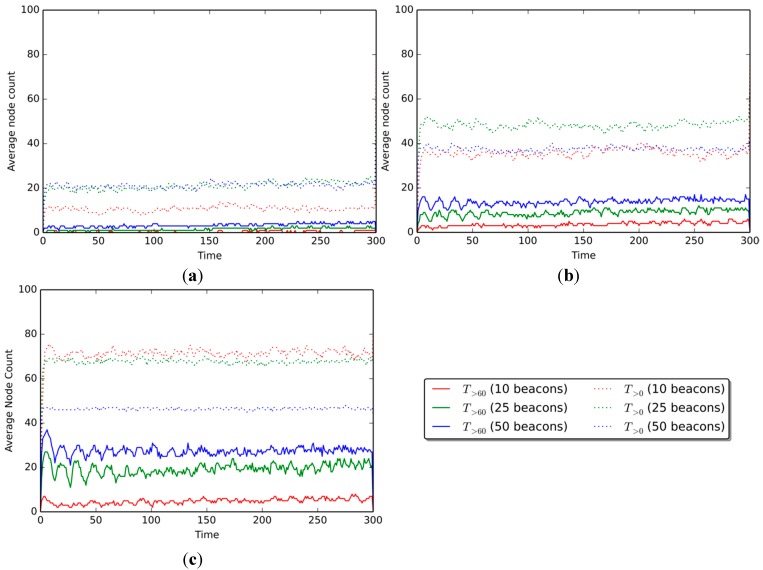
Number of nodes with confidence over the threshold (per number of beacons in scenario) in the ranges of (**a**) 30 m, (**b**) 50 m, and (**c**) 80 m.

Figure 6 shows the number of nodes that know their position with fitness scores over specific thresholds. Results are shown for scenarios with communication ranges of 30, 50, and 80 m. The curves on each graph represent the number of nodes over a given fitness threshold, for each of the three percentages of beacon nodes (10%, 25%, and 50%). The solid curves are for nodes with fitness scores over 60 ($T_{>60}$), and dotted curves are for nodes with fitness scores greater than 0 ($T_{>0}$).

Figure 6a shows results for the scenario with a communication range of 30 m. The average number of nodes with fitness $T_{>60}$ is 0.45 nodes when 10% beacons are present, 1.80 for 25% beacons, and 3.60 for 50% beacons. The quantity of $T_{>0}$ nodes is generally higher, with 10.81, 20.27, and 21.17 nodes on average when 10%, 25%, and 50% beacons are present, respectively.

When the communication range is augmented to 50 m (Figure 6b), the average number of nodes over the threshold increases to 4.05, 9.01, and 13.51 $T_{>60}$ nodes, and 35.59, 37.84, and 48.64 $T_{>0}$ nodes, for the 10%, 25%, and 50% beacon amounts, respectively. Finally, for the 80-m communication range (Figure 6c), the average node count amounts to 4.95, 19.37, and 27.93 $T_{>60}$ nodes, and 46.84, 68.47, and 72.07 $T_{>0}$ nodes for each of the three beacon amounts, respectively.

Results show that the number of nodes over both the $T_{>60}$ and the $T_{>0}$ fitness thresholds increases as the communication range of the devices is augmented, as expected. This increase is smaller when the threshold is greater (e.g., 60), but more noticeable when the threshold is lower (e.g., 20). When 10% beacon nodes are present in a scenario, there are +1.35 $T_{>60}$ nodes on average when the communication range is augmented from 30 to 50 m (Figure 6a,b), and +1.80 when going from 50 to 80 m (Figure 6b,c). The $T_{>0}$ node number behaves in a similar way, with +9.46 and +0.90 nodes when increasing the communication range from 30 to 50 m, and from 50 to 80, respectively.

For the 25% beacon scenario, the $T_{>60}$ nodes show an increase of 4.95 and 4.50 nodes on average when the communication range is augmented from 30 to 50 m (Figure 6a,b), and then from 50 to 80 ms (Figure 6b,c), respectively. Meanwhile, $T_{>0}$ nodes show an increase of 2.25 and 10.81 nodes, going from a communication range of 30 to 50 m, and from 50 to 80 m. Finally, when 50% nodes are present in the scenario, the number of $T_{>60}$ nodes increases by 14.41 and 8.56, while the $T_{>0}$ node amount grows by 21.62 and 3.60 nodes for the corresponding increments in the communication range.

### 5.2. Findings

There is a direct relation between the number of beacon nodes present in the scenario and both the number of nodes that know their position and the fitness scores of such a scenario. Having more beacons increases the likelihood of non-beacons finding neighbors that know their positions (*i.e.*, nodes with fitness scores over $T_{>0}$). This allows more non-beacons to estimate their positions, and allows for higher average fitness scores in the scenario.

There is also a direct relation between the number of beacon nodes present the scenario and the energy consumption. Beacon nodes carry an additional cost associated with using a positioning strategy, namely establishing and maintaining communication with the provider (e.g., GPS satellites, a centralized server), and activating the required peripherals. Thus, if more beacons are present in a scenario, the overall energy consumption tends to increase. However, the individual energy consumption of non-beacons tends to decrease, because fewer positioning requests are required to obtain meaningful positioning information from their neighbors.

Augmenting the communication range improves the confidence levels of the scenario, but increases the energy consumption. Greater communication ranges allow non-beacons to reach more neighbors when requesting positioning information, facilitating collaboration and thus increasing both the average fitness score of the scenario and the number of nodes that know their positions. However, having a greater range entails greater energy consumption. Given that more nodes are reached by each positioning request, more energy is consumed through communication.

Conversely, lowering the communication decreases the number of nodes that know their position and the confidence of the scenario, and increases the energy consumption. This is because fewer neighbors are reached by non-beacons requesting positioning information. Since non-beacons are less likely to estimate their positions, the number of nodes over $T_{>0}$ and the overall fitness scores tend to decrease. Moreover, since isolated individual nodes are unable to reach viable neighbors, they must expend more energy sending additional positioning requests.

Finally, limiting the communication of devices to a single channel is less energy-demanding than allowing multiple channels. When using a single communication channel (*i.e.*, Wi-Fi), only one positioning request has to be sent; when using multiple channels, a request has to be sent from each of the corresponding peripherals (one per available channel). On the other hand, sending requests through multiple channels implies that some requests will be received more than once by some neighbors, and others will be lost if no neighbors are capable to receive them. This translates into greater energy consumption, due to the additional communications.

### 5.3. Tuning the Model Based on the Findings

**Figure 7:**
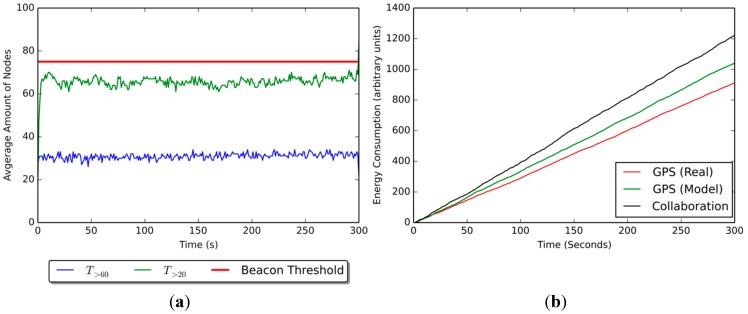
(**a**) Average node count and (**b**) average energy consumption for the scenario.

The model was improved based on our findings, and additional experiments were performed. Figure 7a shows the average number of non-beacons that know their position at a given time (equivalent to Figure 6b), which was obtained during the performance simulations. The communication range has been set to 50 m at maximum, and the number of beacons at 25%. The red line shows the beacon threshold, or the maximum number of non-beacons (*i.e.*, 75). The blue line shows the number of non-beacons with an average fitness over 60; these nodes represent how many nodes know their position with a degree of confidence acceptable for our model. The green line, on the other hand, shows the number of non-beacons with fitness over 20, representing all the nodes that know their position even if it is not dependable, *i.e.*, the accumulated positioning error over time is too great for their position to be accurate.

Results show that, on average, around 50% of the nodes present in the scenario shown on Figure 7a (25 beacons and approximately 25 non-beacons) are able to estimate their position with acceptable confidence, *i.e.*, with decay over 60. In addition, about 35% of non-beacons know their position with decay between 20 and 60, although their positioning information would be of no use for collaboration under our model.

The energy consumption of each device has been represented using an arbitrary unit count. Each of these arbitrary units is associated to an energy cost of around 166±0.04% mW [40] (depending on the type of antenna used), and thus, each time an arbitrary unit is added, it is in fact representing expended energy. This is done to avoid setting a fixed energy value for all nodes, given that not all devices use the same type of peripherals in real-world scenarios. The energy unit count is initialized at 0 at the beginning of the simulations, and increases as time passes, and also each time a peripheral is used either to sense or to communicate.

Figure 7b shows the accumulated energy consumption values (in arbitrary units) in a scenario with 25% beacons and a communication range of 50%. The GPS (Real) curve represents the energy utilization of the GPS in a real-world scenario, while the GPS (Model) curve is the approximation that has been achieved on the simulation. Results from GPS (Real) and GPS (Model) should be identical or at least similar, given that both represent the same variable (the first obtained from literature, the other from the simulation). However, there is a difference of 111 arbitrary units (18,547.54 mW) between these curves, which means the approximation from the simulation is off and must be corrected to provide more accurate values for energy consumption. It is important to note that the Collaboration curve, which represents the energy consumption of a non-beacon node, is unexpectedly 69 arbitrary units (11,571.86 mW) below the goal energy consumption, represented by the GPS (Model) curve.

## 6. Limitations of the Model

This section details the limitations of the proposed model; for each of them we discuss its impact on our research, and possible ways to overcome them in future work.

(1) All of the research has been performed through simulations of outdoor environments, which could be different from a real disaster relief scenario. However, the models used in the simulations are well accepted by the research community; in particular, the mobility model used for the nodes was the most accepted one for disaster scenarios.

(2) The model is focused on outdoor scenarios and positioning strategies, and does not address aspects involved in indoor positioning. Since most indoor positioning systems rely on electricity and infrastructure-based communication networks, which could be damaged or collapsed and thus unavailable, they have not been taken into account. However, given that the model is detached from the actual positioning, indoor strategies could be considered in further iterations of the model if required.

(3) The current version of the positioning model does not address the security and privacy concerns related to the shared information; therefore, it could be used to track users, or even to deceive users with false information. However, the model follows the lines of peer-to-peer (P2P) networks file sharing, which are highly dependent on the user’s intent, but still have been proven to be relatively safe for most of the user’s operations (specifically file sharing). Ensuring the security and privacy of the shared information is a challenge that will be addressed as part of our future work.

## 7. Conclusions and Future Work

This paper presents a context-aware positioning model for outdoor environments, particularly to support disaster relief efforts. The model enables devices to estimate their position based on information gathered from their context, either directly from a positioning system or through collaboration with neighboring devices. This capability is required due to the fact that in disaster relief efforts, at least in developing countries, there is a large variety of mobile computing devices with no capability of self-positioning, but that potentially can be useful to support the response activities. The proposed model also considers the typical features of these working environments; *i.e.*, unavailability of infrastructure-based electrical energy and digital communication, heterogeneity of devices, and instability of the communication link.

The model succeeds in providing a degree of positioning to mobile applications running on devices otherwise unable to position themselves, by sharing positioning information with their neighbors. However, due to the nature of the collaboration (*i.e.*, the reference points’ positions have a variable error), accuracy is significantly lower than using a positioning strategy directly, showing errors ranging from less than 1 m to over 12 m greater than when using GPS. Thus, the estimations need to be performed at shorter time intervals in order to correct eventual inaccurate measurements.

Likewise, by using the set of recommendations assembled by the model, a device is able to access any of the positioning strategies available in its surroundings. This eliminates the need to start the whole positioning process over when there has been no significant change in context, since the device can use any of the recommended strategies, ideally in order of priority. The interval at which the model senses the environment depends on the speed of the device, and on the position accuracy requirement of the consumer application.

In terms of energy, despite advances in battery technology, maintaining an active connection (e.g., GPS or Wi-Fi) consumes significant amounts of power. Thus, the model must make additional decisions to ensure extended operation to the devices, e.g., sensing the context of a device to determine whether it is moving or not, in order to avoid requesting an unnecessary positioning estimation, or determining whether a device has enough energy to go through the positioning process without jeopardizing the device’s basic functions. Although this approach greatly reduces the energy consumption, sensing the context is still energy demanding. This means that an optimal context-sensing interval must be found in order to ensure less energy usage.

Future work involves using a random forest classifier in place of the randomized decision tree that is currently used by the model. The random forest strategy [41] performs very well compared to many other classifiers, including discriminative analysis, support vector machines, and neural networks, and it is robust against over-fitting. We believe this could help improve the recommendations of positioning methods given by the model.

Moreover, we plan to conduct an evaluation of the energy consumption of the proposed model, not only to node level, but also considering the whole interconnected system. Several settings of the application scenario should be simulated and analyzed. The obtained results will help us improve the positioning priorities considered during the strategy choosing process performed by the model. Security and privacy issues related to the shared information are also some of the challenges to address during the next step.

## References

[1] Ochoa S.F., Pino J.A. (2008). Challenges for Decision Support in Urban Disaster Scenarios.

[2] Ochoa S.F., Santos R. (2015). Human-centric Wireless Sensor Networks to Improve Information Availability During Urban Search and Rescue Activities. Inf. Fusion.

[3] Dourandish B., Zumel N., Manno M. Command and control during the first 72 hours of a joint military-civilian disaster response. Proceedings of the Command and Control Research and Technology Symposium.

[4] Ochoa S.F., Neyem A., Pino J.A., Borges M. (2007). Supporting group decision making and coordination in urban disasters relief efforts. J. Decis. Syst..

[5] Manoj B.S., Baker A. (2007). Communication challenges in emergency response. Commun. ACM.

[6] Schöning J., Rohs M., Krüger A., Stasch C. (2009). Improving the communication of spatial information in crisis response by combining paper maps and mobile devices. Mobile Response.

[7] Braunstein B., Trimble T., Mishra R., Manoj B.S., Lenert L., Rao R. Challenges in using distributed wireless mesh networks in emergency response. Proceedings of the ISCRAM.

[8] Mendonça D. (2007). Decision support for improvisation in response to extreme events: learning from the response to the 2001 world trade center attack. Decis. Support Syst..

[9] Bradler D., Schiller B. Towards a distributed crisis response communication system. Proceedings of the ISCRAM.

[10] Federal Communications Commission (FCC) (2011). The Role of Deployable Aerial Communications Architecture in Emergency Communications and Recommended Next Steps.

[11] Rodríguez-Covili J.F., Ochoa S.F., Pino J.A., Messeguer R., Medina E., Royo D. (2001). A communication infrastructure to ease the development of mobile collaborative applications. J. Netw. Comput..

[12] Fitrianie S., Rothkrantz L. Dynamic Routing during Disaster Events. Proceedings of the ISCRAM.

[13] Shin B.J., Lee K.W., Choi S.H., Kim J.Y., Lee W.J., Kim H.S. Indoor WiFi Positioning System for Android-based Smartphone. Proceedings of the International Conference on Information And Communication Technology Convergence 2010.

[14] Hofmann-Wellenhof B., Lichtenegger H., Wasle E. (2008). GNSS—Global Navigation Satellite Systems: GPS, GLONASS, Galileo, and More.

[15] Huang B., Yao Z., Cui X., Lu M. Dilution of Precision Analysis for GNSS Collaborative Positioning. IEEE Trans. Veh. Technol..

[16] Zhang P., Zhao Q., Li Y., Niu X., Zhuang Y., Liu J. (2015). Collaborative WiFi fingerprinting using sensor-based navigation on smartphones. Sensors.

[17] Sahoo P.K., Hwang I.S. (2011). Collaborative localization algorithms for wireless sensor networks with reduced localization error. Sensors.

[18] Savarese C., Rabaey J.M., Langendoen K. Robust Positioning algorithms for distributed ad-hoc wireless sensor networks. Proceedings of the General Track of the Annual Technical Conference on USENIX 2002.

[19] Čapkun S., Hamdi M., Hubaux J.P. (2002). GPS-free positioning in mobile *ad hoc* networks. Clust. Comput..

[20] Shaw B., Shea J., Sinha S., Hogue A. Learning to rank for spatiotemporal search. Proceedings of the 6th ACM International Conference on Web Search and Data Mining.

[21] Eltahir I.K. The impact of different radio propagation models for mobile ad hoc networks (manet) in urban area environment. Proceedings of the 2nd International Conference on Wireless Broadband and Ultra Wideband Communications.

[22] Camp T., Boleng J., Davies V. (2002). A survey of mobility models for ad hoc network research. Wirel. Commun. Mob. Comput..

[23] Stoffers M., Riley G. Comparing the ns-3 propagation models. Proceedings of the IEEE 20th International Symposium on Modeling, Analysis & Simulation of Computer and Telecommunication Systems (MASCOTS).

[24] Oliver M., Badland H.M., Mavoa S., Duncan M.J., Duncan J.S. (2010). Combining GPS, GIS, and Accelerometry: Methodological Issues in the Assessment of Location and Intensity of Travel Behaviors. J. Phys. Act. Health.

[25] Jacquet P., Muhlethaler P., Clausen T., Laouiti A., Qayyum A., Viennot L. Optimized link state routing protocol for ad hoc networks. Proceedings of the IEEE International Multi-Topic Conference (INMIC 2001): Technology for the 21st Century 2001.

[26] Rodríguez-Covili J.F., Ochoa S.F., Pino J.A. (2012). High Level MANET Protocol: Enhancing the Communication Support for Mobile Collaborative Work. J. Netw. Comput. Appl..

[27] Hall D.L., Llinas J. (1997). Introduction to multisensor data fusion. Proc. IEEE.

[28] Beauregard S., Haas H. Pedestrian dead reckoning: A basis for personal positioning. Proceedings of the 3rd Workshop on Positioning, Navigation and Communication.

[29] Vera R., Ochoa S.F., Aldunate R.G. (2011). EDIPS: An Easy to deploy indoor positioning system to support loosely coupled mobile work. Pers. Ubiquitous Comp..

[30] Hightower J., Borriello G. (2001). Location systems for ubiquitous computing. IEEE Comput..

[31] Barequet G., Har-Peled S. (2001). Efficiently approximating the minimum-volume bounding box of a point set in three dimensions. J. Algorithms.

[32] Henderson T.R., Roy S., Floyd S., Riley G.F. Ns-3 Project Goals. Proceedings of the ACM 2006 Workshop on ns-2: The IP Network Simulator.

[33] Aschenbruck N., Ernst R., Gerhards-Padilla G., Schwamborn M. BonnMotion: A mobility scenario generation and analysis tool. Proceedings of the 3rd International ICST Conference on Simulation Tools and Techniques 2010.

[34] Aschenbruck N., Gerhards-Padilla E., Martini P. (2009). Modeling mobility in disaster area scenarios. Perform. Eval..

[35] Lee K., Hong S., Kim S.J., Rhee I., Chong S. Slaw: A new mobility model for human walks. Proceedings of INFOCOM 2009.

[36] Hong X., Gerla M., Pei G., Chiang C.C. A group mobility model for *ad hoc* wireless networks. Proceedings of the 2nd ACM International Workshop on Modeling, Analysis and Simulation of Wireless and Mobile Systems 1999.

[37] Bianchi G. (2000). Performance analysis of the IEEE 802.11 distributed coordination function. IEEE J. Sel. Areas Commun..

[38] Medeisis A., Kajackas A. On the use of the universal Okumura-Hata propagation prediction model in rural areas. Proceedings of the IEEE 51th Vehicular Technology Conference 2000.

[39] Lassabe F., Canalda P., Chatonnay P., Spies F., Baala O. A Friis-based calibrated model for WiFi terminals positioning. Proceedings of the 6th IEEE International Symposium on a World of Wireless Mobile and Multimedia Networks.

[40] Carroll A., Heiser G. An analysis of power consumption in a smartphone. Proceedings of USENIX 2010.

[41] Liaw A., Wiener M. (2002). Classification and regression by RandomForest. R News.

